# A U.K. Multicenter Retrospective Study of the Learning Curve and Relative Impact on Success Rates and Procedural Metrics of the RHYTHMIA HDx™ Mapping System

**DOI:** 10.19102/icrm.2023.14054

**Published:** 2023-05-15

**Authors:** Alexander Bates, Mohammad Naseer, Mark Taylor, Nathan Denham, Arthur Yue, Moloy Das, Gwilym M. Morris, Waqas Ullah

**Affiliations:** ^1^University Hospital Southampton NHS Foundation Trust, Southampton, UK; ^2^School of Human Development and Health, Faculty of Medicine, University of Southampton, Southampton, UK; ^3^Freeman Hospital, The Newcastle upon Tyne Hospitals NHS Foundation Trust, Newcastle upon Tyne, UK; ^4^Division of Cardiovascular Sciences, School of Medicine, Faculty of Biology Medicine and Health, University of Manchester, Manchester Academic Health Science Centre, Manchester, UK; ^5^Department of Cardiology, Manchester University Foundation Trust, Manchester Academic Health Science Centre, Manchester, UK

**Keywords:** Ablation, electroanatomic mapping, learning curve, RHYTHMIA HDx™

## Abstract

The learning curve for the novel RHYTHMIA HDx™ 3-dimensional electroanatomic system is unknown. Retrospective data collection was carried out at 3 U.K. centers from the introduction of RHYTHMIA HDx™ (Boston Scientific, Marlborough, MA, USA) and associated mapping and ablation catheters. Patients were matched with controls using the CARTO^®^ 3 mapping system (Biosense Webster Inc., Diamond Bar, CA, USA). Fluoroscopy, radiofrequency ablation, and procedure times; acute and long-term success; and complications were assessed. A total of 253 study patients along with 253 controls were included. Significant correlations existed between procedural efficiency metrics and center experience for de novo atrial fibrillation (AF) ablation (procedure time, Spearman’s ρ = −0.624; ablation time, ρ = −0.795; both *P* < .0005) and de novo atrial flutter (AFL) ablation (ablation time, ρ = −0.566; fluoroscopy time, ρ = −0.520; both *P* = .001). No correlations existed for other assessed atrial arrhythmias. For de novo AF and AFL, metrics significantly improved after 10 procedures in each center (procedure time [AF only, *P* = .001], ablation time [AF, *P* < .0005; AFL, *P* < .0005], and fluoroscopy time [AFL only, *P* = .0022]) and became comparable to those of controls. Acute success and long-term success did not experience significant improvements with experience, but they were comparable to the control group throughout. Complications with RHYTHMIA HDx™ were comparable to those associated with CARTO^®^ 3. In conclusion, a short learning curve exists with the use of RHYTHMIA HDx™ for standardized procedures (de novo AF/AFL). Procedural performance improved and became comparable to that seen with CARTO^®^ 3 following 10 cases at each center. Clinical outcomes at 6 and 12 months and complications were no different from those observed in controls.

## Introduction

The advent of 3-dimensional (3D) electroanatomic mapping systems has led to advances in the visualization of arrhythmic substrate and activation patterns. RHYTHMIA HDx™ (Boston Scientific, Marlborough, MA, USA) is a relatively novel ultra–high-density mapping system with first-documented clinical use in 2015.^1^ The system integrates with the basket-shaped, 64-electrode ORION™ mapping catheter (Boston Scientific).^2,3^ No other mapping system currently has a comparable mapping catheter. RHYTHMIA HDx™ has been used successfully clinically across several arrhythmias^4^ and was shown to be clinically accurate and effective to use with minimal associated complications in a recent prospective trial.^5^

Cardiac ablation requires a high level of technical proficiency. Procedures can be challenging, and efficiency has been demonstrated to improve with experience.^6^ New ablation technology has been shown to possess learning curves in fluoroscopy time, procedure time, and the rate of complications.^7,8^ With the expertise required in ablation, a similar learning curve would be expected with the use of RHYTHMIA HDx™. In this study, our objective was to examine the improvements in procedural efficiency, long-term success, and safety with experience using RHYTHMIA HDx™ for atrial arrhythmia ablation across 3 high-volume ablation centers in the United Kingdom. Our comparator for the study was the well-established CARTO^®^ 3 mapping system (Biosense Webster Inc., Diamond Bar, CA, USA) to enable a retrospective comparison of procedural and outcome measures with a benchmark.

## Materials and methods

### Patient selection

Data were retrospectively collected for 253 consecutive adult patients who had undergone an atrial ablation procedure from the time of the introduction of RHYTHMIA HDx™ at 3 high-volume centers across the United Kingdom from June 2016 to March 2020. This was a retrospective study involving data collection and analysis; hence, ethical approval was not required. Patients were matched with controls who had undergone ablation for the same arrhythmia using the CARTO^®^ 3 mapping system. The matching was performed on a temporal basis, with the control procedure performed closest in time to the equivalent RHYTHMIA HDx™ procedure being submitted. Operators were all highly experienced in the use of CARTO^®^ 3 but had no prior experience with RHYTHMIA HDx™. The procedures included de novo atrial fibrillation (AF), redo AF, de novo atrial tachycardia (AT), redo AT, de novo atrial flutter (AFL), and redo AFL. For the purposes of this study, AFL was considered to be cavotricuspid isthmus (CTI)-dependent. AT could be focal or re-entrant but was non–CTI-dependent.

Assessment of left atrial size was performed by transthoracic echocardiography using either atrial diameter, area, or volume according to local departmental preference.

### Ablation procedure

Procedures were performed according to the local guidelines under conscious sedation or general anesthetic. All RHYTHMIA HDx™ cases used mapping and ablation catheters from Boston Scientific. All mapping using RHYTHMIA HDx™ was performed using the 64-electrode ORION™ catheter. Prior to the introduction of DirectSense (DS) technology, ablation was performed using either the IntellaNav open-irrigated catheter or the IntellaNav MiFi open-irrigated catheter (Boston Scientific). Following its introduction, the IntellaNav MiFi open-irrigated catheter with integrated DS was used. All mapping and ablation catheters used for procedures with CARTO^®^ 3 were manufactured by Biosense Webster.

For de novo AF procedures, a similar approach was taken across centers involving pulmonary vein isolation (PVI), and, for de novo AFL procedures, involving the creation of a CTI line. Additional ablation for these procedures beyond this was done at the discretion of the operator.

Intracardiac echocardiography is not performed as a standard in the United Kingdom and was not used in any cases in this study.

### Study endpoints

Patients were divided by their procedure type and had metrics analyzed within these groups. Procedural efficiency was assessed by analysis of total fluoroscopy, radiofrequency ablation, and procedure times as well as termination of the arrhythmia by the end of the procedure where relevant. Long-term freedom from arrhythmia at 6 and 12 months was assessed from documentation at routine patient follow-up. Where symptoms were recorded, the presence of arrhythmia recurrence was documented from either a 12-lead electrocardiogram or prolonged ambulatory monitoring. Confirmation of complications of the procedure was obtained from procedure reports and hospital documentation.

The learning curve was assessed by comparing 2 phases, the first during which procedural performance markers were improving (P1) and the second occurring when a plateau had been reached (P2). The threshold between these phases was determined visually from a scatterplot and comparison with the control group. An analysis of variance (ANOVA) was performed between P1, P2, and the control group for validation of the threshold.

### Statistics

Statistics were calculated using IBM SPSS Statistics (version 27; IBM Corp., Armonk, NY, USA). A *P* value of <.05 was considered statistically significant. For discrete variables, absolute values and proportions are reported. Continuous data are expressed as mean ± standard deviation values. Bivariate correlations were assessed using Spearman’s rank correlation if both variables were continuous and using point biserial correlation if a variable was dichotomous. The normality of data was assessed using the Shapiro–Wilk test. Multiple independent samples were compared by Welch ANOVA with a post hoc Games–Howell test used to assess for significance within a group. This was due to uneven variances between compared groups as determined by Levene’s test. A chi-squared or Fisher’s exact test was used to test for significance between dichotomous variables.

## Results

### Patient and procedure characteristics

Baseline patient and procedural information is displayed in **Tables 1–3**. Hypertension was the only characteristic with a significant difference in prevalence, being greater in the CARTO^®^ 3 group. Unbalanced patient numbers within procedure groups were caused by either the lack of a suitable control (more RHYTHMIA HDx™ cases than CARTO^®^ 3 cases) or the inclusion of an additional control as compensation (more CARTO^®^ 3 cases than RHYTHMIA HDx™ cases). Contributing patient numbers from each center were 188, 80, and 238, respectively.

### Procedural efficiency

To establish the improvement in procedural efficiency, correlations with center experience were examined. Procedures with a widely accepted standard protocol (PVI for de novo AF and CTI ablation for de novo AFL) showed improvements in multiple metrics with center experience. With increasing center experience, significant improvements were seen for de novo AF in procedure times (Spearman’s ρ = −0.624, *P* < .0005) and radiofrequency ablation times (ρ = −0.795, *P* < .0005), whilst de novo AFL cases had improvements in fluoroscopy times (ρ = −0.520, *P* < .001) and radiofrequency ablation times (ρ = −0.566, *P* < .001). Procedures where the ablation strategy was tailored upon the operative findings (de novo AT, all redo procedures) showed no correlations with these markers.

**Figure 1:**
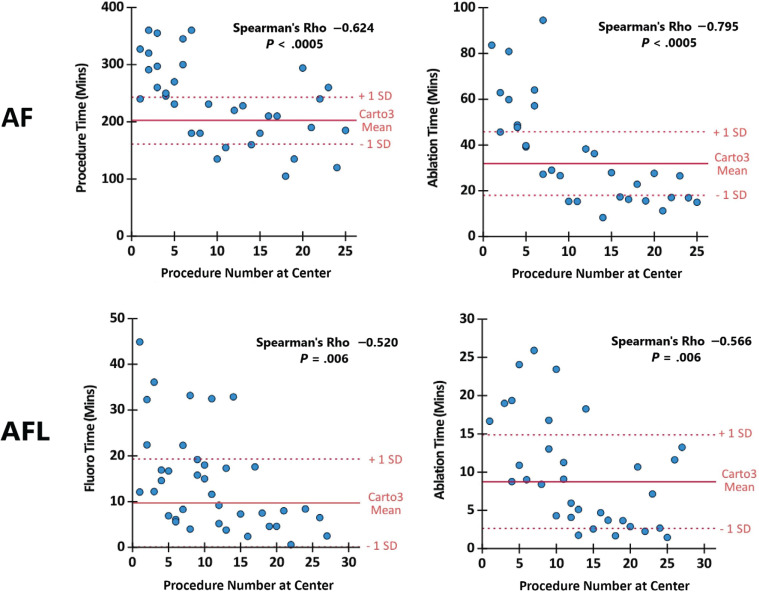
Scatterplots of procedural efficiency metrics against experience for de novo atrial fibrillation and de novo atrial flutter cases. Red lines indicate mean ± standard deviation values of the control group treated with CARTO^®^ 3. *Abbreviations:* AF, atrial fibrillation; AFL, atrial flutter.

Reflecting this, the data for de novo AF and AFL were examined in a scatterplot to establish the presence of a learning curve and the threshold between P1 and P2. Learning curves were seen in all instances, and a threshold was noted at approximately 10 cases **(Figure 1)**.

**Figure 2:**
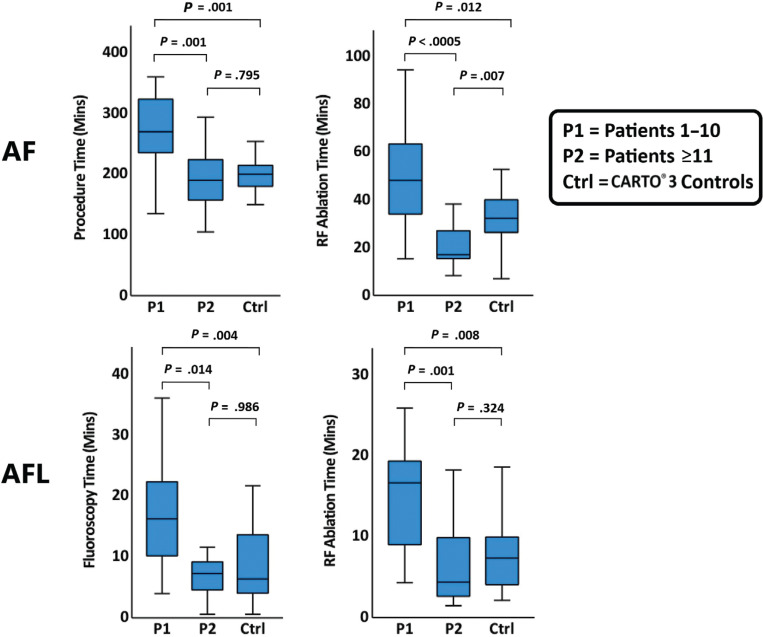
Boxplots comparing procedural efficiency metrics between experience with RHYTHMIA HDx™ and CARTO^®^ 3 for de novo atrial fibrillation and de novo atrial flutter procedures. *Abbreviations:* AF, atrial fibrillation; AFL, atrial flutter; RF, radiofrequency.

To test the validity of this threshold, Welch’s ANOVA with a post hoc Games–Howell test was performed between P1, P2, and the control group **(Figure 2)**. For de novo AF, there was a significant difference between P1, P2, and the control group for procedure time (*P* = .001) and ablation time (*P* < .0005). Post hoc Games–Howell analysis revealed that the decrease in procedure time was significant between P1 and P2 (79.7 min; 95% confidence interval [CI], 30.1–129.3 min; *P* = .001) and between P1 and the control group (69.7 min; 95% CI, 28.4–110.9 min; *P* = .001), but not between P2 and the control group (*P* = .795). The decrease in ablation time was significant between P1 and P2 (30.6 min; 95% CI, 15.3–45.8 min; *P* < .0005), between P1 and the control group (19.5 min; 95% CI, 4.0–35.0 min; *P* = .012), and—interestingly—between the control group and P2 (11.0 min; 95% CI, 2.7–19.4 min; *P* = .007).

For de novo AFL, there was a signficant difference between P1, P2, and the control group for fluoroscopy time (*P* = .004) and ablation time (*P* = .001). Post hoc Games–Howell analysis revealed that the decrease in fluoroscopy time was significant from P1 to P2 (9.3 min; 95% CI, 1.7–16.9 min; *P* = .014) and between P1 and the control group (9.7 min; 95% CI, 3.0–16.3 min; *P* = .004), but not between P2 and the control group (*P* = .986). For ablation time, the decrease was significant from P1 to P2 (9.2 min; 95% CI, 3.7–14.7 min; *P* = .001) and between P1 and the control group (7.2 min; 95% CI, 1.9–12.6 min; *P* = .008), but not between P2 and the control group (*P* = .324).

Procedural efficiency markers for tailored procedures were comparable to those of the control group in all but a single instance where, for redo AF, the fluoroscopy time was significantly shorter using RHYTHMIA HDx™ than when using CARTO^®^ 3 (16.0 ± 8.3 vs. 19.3 ± 10.0 min, *P* = .02).

### Clinical outcomes

**Figure 3:**
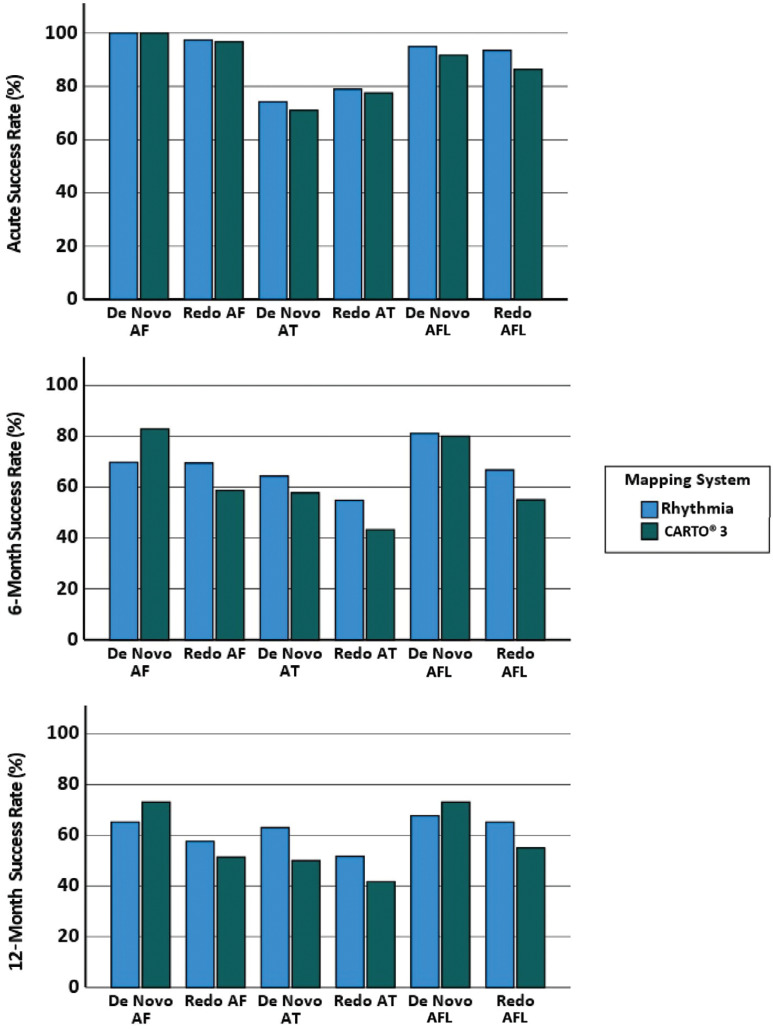
Bar charts comparing acute, 6-, and 12-month success rates of atrial procedures. No significant differences existed between RHYTHMIA HDx™ and CARTO^®^ 3 for success at any time point. *Abbreviations:* AF, atrial fibrillation; AFL, atrial flutter; AT, atrial tachycardia.

Acute success rates for procedures using RHYTHMIA HDx™ were high for all arrhythmias **(Figure 3)**. There were no significant correlations found between experience and acute, 6-, or 12-month freedom from arrhythmia. There were no significant differences in clinical outcomes between RHYTHMIA HDx™ and CARTO^®^ 3 for any procedure type or collectively.

### Complications

A total of 8 (3.2%) complications were noted with the use of RHYTHMIA HDx™. The most frequent complication was groin hematoma related to vascular access. Two pericardial effusions related to ablation occurred, necessitating pericardiocentesis and placement of a drain. All drains were removed after 24 h, and no further intervention was required. One pericardial effusion was detected on a routine post-procedural transthoracic echocardiogram and did not require intervention. In the CARTO^®^ 3 group, 1 patient experienced a cardiac arrest due to cardiac tamponade from a tear in the coronary sinus. This was repaired by emergency cardiac surgery and resulted in an anoxic brain injury. A single instance of complete atrioventricular block occurred during a redo AFL case during ablation of the CTI line. The patient received a permanent pacing device the following day. No thromboembolic complications occurred. There was no significant difference in the complication rate between mapping systems (*P* = .400) or correlation with the experience with RHYTHMIA HDx™ (*P* = .832). There were no procedure-related deaths. Complications are detailed in **Table 4**.

## Discussion

This aim of this study was to assess for and examine the learning curve for using a new 3D electroanatomic mapping system.

Improvements in procedural efficiency were found for de novo AF and AFL ablation, which are both procedures that lend themselves most easily to assessment as they entail standardized ablation strategies (PVI and CTI ablation). This contrasts with the tailored approach necessary for treating our other investigated arrhythmias.

For both de novo AF and AFL, there was a significant reduction in ablation time from P1 to P2. For AFL, this is likely explained by a combination of increased experience and the contemporaneous introduction to the market of DS technology at the P1–P2 threshold. With increased experience, fewer and more accurate ablation lesions could be applied, whilst, with DS, ablation lesions could be curtailed when a target or plateau in local impedance (LI) drop was achieved. For de novo AF, all procedures were performed with DS and prior to the publication of LI targets in the Electrical Coupling Information from the RHYTHMIA HDx™ System and DirectSense Technology in Subjects with Paroxysmal Atrial Fibrillation (LOCALIZE) study.^9^ This leaves only increases in skill and experience to explain the decrease in ablation time. The same reasoning is likely to explain the reduction in procedure time between P1 and P2 for de novo AF.

Interestingly, there was a significant reduction in fluoroscopy time with increased experience with RHYTHMIA HDx™ for de novo AFL but not de novo AF. Ablation of the CTI during AFL ablation is commonly performed under fluoroscopic guidance and without 3D mapping assistance. Consequently, operators are experienced in fluoroscopic catheter positioning to complete a successful CTI line. The reduction in fluoroscopy time may reflect an operator’s increasing confidence in the accuracy of RHYTHMIA HDx™, whereby ablation catheter position becomes checked less frequently with experience. In contrast, for de novo AF procedures, fluoroscopy is typically used for reference catheter positioning into the coronary sinus and to assist with transseptal puncture, which are both maneuvers that would not use the mapping system as standard.

The learning curves seen for de novo AF and AFL were short, requiring only 10 procedures per center to become comparable to those associated with CARTO^®^ 3. This suggests that, although some familiarity with a new mapping system is necessary, the underlying procedural accuracy and manual skills required are highly transferable.

In a certain instance, RHYTHMIA HDx™ showed an improved efficiency over CARTO^®^ 3. For de novo AF, RHYTHMIA HDx™ had a significantly shorter ablation time in P2 than in P1 and compared to CARTO^®^ 3. While the difference in ablation time between P1 and P2 is likely due to improved procedural accuracy, one would not expect this to extend beyond the CARTO^®^ 3 control group. A possible explanation for this is the difference in ablation targets between these mapping systems. In these RHYTHMIA HDx™ cases, a DS catheter was used, allowing ablations to be tailored based on LI.^10^ Conversely, CARTO^®^ 3 uses a composite score of contact force, time, and power called the Ablation Index,^11,12^ which is a parameter that is pre-set for locations in the PVI line. The results suggest that LI-guided ablation has a shorter ablation time per lesion than the Ablation Index, likely due to curtailing of ablations in response to an adequate tissue response to ablation.

Despite the presence of a learning curve in efficiency for some of the procedures performed with RHYTHMIA HDx™, the clinical outcomes observed acutely and at 6 and 12 months were in line with those of the CARTO^®^ 3 control group and results from a recent meta-analysis.^4^ The number of complications observed using RHYTHMIA HDx™ was low (3.2%), comparable to that associated with CARTO^®^ 3 and found in other published studies,^4^ and the complications were mainly unrelated to use of the system. This is important as it suggests that using the new system, while associated with a learning curve, is not associated with a disadvantage to the patient with regard to safety or clinical success. The value of using RHYTHMIA HDx™ over a different system therefore would be in specific operator preference for ultra–high-density mapping or DS technology.

### Previous studies

Rottner et al.^13^ performed a learning curve analysis as part of their study comparing procedural and clinical outcome data between RHYTHMIA HDx™ and CARTO^®^ 3 for patients undergoing AF ablation. The authors divided their 37 RHYTHMIA patients into 2 groups, observing a significant reduction in procedure time due to quicker mapping with the ORION™ catheter but no other metrics. Likewise, our study also showed that experience leads to a reduction in not only procedure time but also ablation time, which plateaued notably lower than in the patient cohort of Rottner et al. (45.5 vs. 20.8 min). The disparity between studies is likely due to the difference in ablation catheter use and the advent of DS technology between them. While all our de novo AF ablation patients received LI-guided ablation, this was not the case in the study by Rottner et al.

Several studies have compared procedural markers between CARTO^®^ 3 and RHYTHMIA HDx™ for AF, as summarized in a recent meta-analysis.^4^ There were no significant differences in procedure times, but 2 studies noted a reduction in ablation time, similar to our findings.^14,15^ There were contrasting results for fluoroscopy, with 1 study finding a reduction with RHYTHMIA HDx™,^16^ 1 finding a reduction with CARTO^®^ 3,^13^ and 1 reporting no significant difference.^15^ Acute procedural success was 100% in almost all studies. Pooled 6- and 12-month clinical outcomes were not significantly different between RHYTHMIA HDx™ and CARTO^®^ 3 procedures (80.3%/71.6% vs. 73.8%/65.7%). Complication rates associated with RHYTHMIA HDx™ were low (0%–8.1%) across all studies, which, when considered in combination with our study, affirms RHYTHMIA HDx™ as a safe mapping system to use.^4^ Our study complements these results, expands on them to look at all atrial ablation types, and also further investigated the learning curves involved with the adoption of this new system for experienced operators.

The Prospective Registry on User Experience with the RHYTHMIA HDx™ Mapping System for Ablation Procedures (TRUE-HD) study^5^ was the first prospective evaluation of RHYTHMIA HDx™ in terms of performance and safety. The authors found RHYTHMIA HDx™ to be clinically effective, with a combined acute procedural success rate of 83.3%, a value similar to that from our study. Likewise, the overall complication rate was low at 4%, with a device-related complication rate of only 0.57%. The TRUE-HD study did not follow up with patients beyond their procedure and did not enroll a control group. Our study, although retrospective, compared outcomes to a matched group who had their procedures performed using CARTO^®^ 3 and found both clinical effectiveness and safety to be comparable.

### Limitations

To assess learning curves, the number of procedures was stratified by center and not operator, which would have been preferable. This was to avoid over-dilution of the data. To overcome this limitation, repeating this study during a larger time period with more centers enrolled would be recommended.

Data were collected retrospectively; thus, analysis was limited to the procedural markers discussed. Other markers to assess the procedural efficiency of RHYTHMIA HDx™, such as the number of mapping points and mapping time, were not readily available and could not be analyzed, which would have been useful as a direct representation of experience with the ORION™ mapping catheter. Similarly, the number of radiofrequency ablations performed at each procedure to assess improvements and efficiency with RHYTHMIA HDx™–compatible ablation catheters was not available.

Procedure time was from the beginning to the end of the procedure, regardless of time spent using RHYTHMIA HDx™ itself, although this would be expected to be fairly consistent from procedure to procedure. Additionally, data regarding the use of single or dual transseptal punctures were not available and may have affected procedural metrics; however, they are not anticipated to be different between P1, P2, and the control group.

The comparison with the control group using CARTO^®^ 3 was a retrospective, non-randomized comparison and can only be considered hypothesis-generating.

Ideally, the effect of the introduction of DS upon procedural efficiency metrics should have been analyzed, but the division of data between pre- and post-DS for de novo AF was uneven (pre-DS, 0; post-DS, 35), and that for AFL resembled the division between P1 and P2. Consequently, the data did not lend themselves to a detailed statistical analysis.

Finally, the clinical follow-up reflected practice in the United Kingdom, which is focused on symptom recurrence and a limited number of electrocardiogram recordings. To assess arrhythmic recurrence in a more detailed fashion, a preferred method would have been prolonged electrocardiogram monitoring in a prospective study.

## Conclusions

Improvements in procedural efficiency are seen with experience using RHYTHMIA HDx™ with standardized ablation strategies. The learning curve is short, with only 10 procedures required per center to see significant improvements. Clinical outcomes and complication rates are acceptable and no different from the well-established CARTO^®^ 3 mapping system, suggesting no safety or efficacy disadvantage to the adoption of the newer system, despite the presence of a learning curve.

## Figures and Tables

**Table 1:** Baseline Clinical Characteristics

	All Patients	RHYTHMIA HDx™	CARTO^®^ 3	*P* Value
n	506	253	253	
Age (years)	62.4 ± 12.8	62.3 ± 13.1	62.6 ± 12.5	.805
Male sex	333 (65.8%)	167 (66.0%)	166 (65.6%)	.925
Comorbidities				
Coronary artery disease	88 (17.4%)	39 (15.4%)	49 (19.4%)	.241
Valvular heart disease	80 (15.8%)	38 (15.0%)	42 (16.6%)	.626
Heart failure	114 (22.5%)	54 (21.3%)	60 (23.7%)	.523
HFREF	66	32	34	.792
HFmREF	22	10	12	.827
HFPEF	26	12	14	.840
Cerebrovascular disease	32 (6.3%)	16 (6.3%)	16 (6.3%)	1.000
Hypertension	211 (41.7%)	93 (36.8%)	118 (46.6%)	.024
Diabetes mellitus	65 (12.8%)	37 (14.6%)	28 (11.1%)	.232
COPD	24 (4.7%)	13 (5.1%)	11 (4.3%)	.676
Obstructive sleep apnea	8 (1.6%)	3 (1.2%)	5 (2.0%)	.476
eGFR	76.5 ± 16.6	77.9 ± 15.0	75.1 ± 18.0	.059
Left atrial size				
Normal	257 (61.2%)	125 (61.0%)	132 (61.4%)	.310
Mild	85 (20.2%)	45 (22.0%)	40 (18.6%)	
Moderate	39 (9.3%)	14 (6.8%)	25 (11.6%)	
Severe	39 (9.3%)	21 (10.2%)	18 (8.4%)	
LV ejection fraction (%)	51.6 ± 8.8	51.6 ± 8.3	51.5 ± 9.2	.878

*Abbreviations:* COPD, chronic obstructive pulmonary disease; eGFR, estimated glomerular filtration rate; HFmREF, heart failure with moderately reduced ejection fraction; HFPEF, heart failure with preserved ejection fraction; HFREF, heart failure with reduced ejection fraction; LV, left ventricular. Cerebrovascular disease was defined as previous stroke or transient ischemic attack.

**Table 2:** Procedure Types Undertaken for RHYTHMIA HDx™ and CARTO^®^ 3

	All Patients	RHYTHMIA HDx™	CARTO^®^ 3
De novo AF	67 (13.2%)	35 (13.8%)	32 (12.6%)
Paroxysmal	38 (7.5%)	22 (8.7%)	16 (6.3%)
Persistent	29 (5.7%)	13 (5.1%)	16 (6.3%)
Redo AF	170 (33.6%)	78 (30.9%)	92 (36.3%)
Paroxysmal	111 (21.9%)	51 (20.2%)	60 (23.7%)
Persistent	59 (11.7%)	27 (10.7%)	32 (12.6%)
De novo AT	62 (12.3%)	31 (12.3%)	31 (12.3%)
Redo AT	78 (15.4%)	38 (15.0%)	40 (15.8%)
Atrial flutter	76 (15.0%)	40 (15.8%)	36 (14.2%)
Redo atrial flutter	53 (10.5%)	31 (12.3%)	22 (8.7%)
Previous ablations	301 (59.5%)	147 (58.1%)	154 (60.9%)

*Abbreviations:* AF, atrial fibrillation; AT, atrial tachycardia. Absolute values are given. Percentages are of all procedures performed for that mapping system.

**Table 3:** Use of DS Ablation Catheter

	All	Pre-DS	DS
De novo AF	35	0	35
Redo AF	78	23	55
De novo AT	31	9	22
Redo AT	38	21	17
Atrial flutter	40	11	29
Redo atrial flutter	31	12	19

*Abbreviations:* AF, atrial fibrillation; AT, atrial tachycardia; DS, DirectSense. Absolute values are given.

**Table 4:** Complications of 506 Atrial Ablation Procedures

	RHYTHMIA HDx™	CARTO^®^ 3
Complications		
Total	8 (3.2%)	5 (2.0%)
Hematoma	3 (1.2%)	1 (0.4%)
Aortic puncture	1 (0.4%)	0
Pericardial effusion	2 (0.8%)	1 (0.4%)
Anaphylaxis	1 (0.4%)	0
Bradycardia requiring permanent pacing	0	1 (0.4%)
Coronary artery spasm	1 (0.4%)	0
Left atrial appendage dissection	0	1 (0.4%)
Anoxic brain injury	0	1 (0.4%)
